# Alginate Hydrogel Beads with a Leakproof Gold Shell for Ultrasound-Triggered Release

**DOI:** 10.3390/pharmaceutics17010133

**Published:** 2025-01-19

**Authors:** Marcus Flowers, Alex Paulsen, Claire R. W. Kaiser, Adam B. Tuma, Hubert H. Lim, Brenda M. Ogle, Chun Wang

**Affiliations:** 1 Department of Biomedical Engineering, University of Minnesota, 7-105 Hasselmo Hall, 312 Church Street SE, Minneapolis, MN 55455, USA; flowe105@umn.edu (M.F.); paul1139@umn.edu (A.P.); kaise364@umn.edu (C.R.W.K.); hlim@umn.edu (H.H.L.); ogle@umn.edu (B.M.O.); 2 Department of Otolaryngology-Head and Neck Surgery, University of Minnesota, Phillips Wangensteen Building, 516 Delaware Street SE, Suite 8-240, Minneapolis, MN 55455, USA; adam.tuma@northwestern.edu; 3 Institute for Translational Neuroscience, Medical School, University of Minnesota, 420 Delaware Street SE, Minneapolis, MN 55455, USA

**Keywords:** triggered release, ultrasound, hydrogel, alginate, gold

## Abstract

**Background/Objectives:** Focused ultrasound has advantages as an external stimulus for drug delivery as it is non-invasive, has high precision and can penetrate deep into tissues. Here, we report a gold-plated alginate (ALG) hydrogel system that retains highly water-soluble small-molecule fluorescein for sharp off/on release after ultrasound exposure. **Methods:** The ALG is crosslinked into beads with calcium chloride and layered with a polycation to adjust the surface charge for the adsorption of catalytic platinum nanoparticles (Pt NPs). The coated bead is subject to electroless plating, forming a gold shell. Ultrasound is applied to the gold-plated ALG beads and the release of fluorescein with or without ultrasound stimulation is quantified. **Results:** Polyethylenimine (PEI), not poly-L-lysine (PLL), is able to facilitate Pt NP adsorption. Gold shell thickness is proportional to the duration of electroless plating and can be controlled. Gold-plated ALG beads are impermeable to the fluorescein cargo and have nearly zero leakage. Exposure to focused ultrasound initiated the release of fluorescein with full release achieved after 72 h. **Conclusions:** The gold-plated ALG hydrogel is a new material platform that can retain highly water-soluble molecules with a sharp off/on release initiated by focused ultrasound.

## 1. Introduction

The field of drug delivery has evolved significantly, with triggerable drug release systems emerging as a promising strategy for achieving precise spatiotemporal control over drug release [1,2]. Systemically delivered drugs circulate throughout the body, leading to suboptimal pharmacokinetics and increased side effects [3]. In contrast, triggerable drug delivery systems (DDSs) can enhance drug efficacy and safety with precisely controlled, on-demand, localized release. This minimizes required dosages and attenuates the unwanted side effects associated with off-target delivery [1]. Reducing the risks of off-target delivery expands the range of viable therapeutics and enables more complex pharmaceutical strategies.

There is a persistent need for precise temporal control over initiating drug release in both clinical settings and benchtop science. For example, temporally controlled/delayed drug delivery is important in the post-operative care of ophthalmic diseases such as cataract and macular degeneration [4,5]. Stem cell-based tissue engineering relies on protocols with extremely precise windows of drug exposure to achieve robust cell differentiation. One example is a well-studied differentiation protocol demonstrating consistent cardiomyocyte specification almost exclusively through the acute delivery of IWP-2, a Wnt pathway inhibitor [6]. Therefore, establishing control over drug delivery timing with triggered release strategies will help advance multiple fields in medicine.

Both endogenous and exogenous triggers are being explored for triggered release DDSs [7]. Endogenous triggers include enzymatic, pH, and redox conditions, which may vary with the physiological and pathological state of patients. On the other hand, exogenous or external triggers, such as light, electric and magnetic fields, are particularly useful because they can be imposed at will. One example is a near-infrared-light-activated micellular system for delivering a hydrophobic cancer drug that, combined with the photothermal effect, induces an efficient antitumor response [8]. However, photothermal systems like this have a limited ability to reach and actuate drug release within deep tissue layers.

Among exogenous triggers, ultrasound has garnered considerable interest because of its non-invasive nature and its ability to penetrate deep into tissues [9]. Ultrasonic waves can be focused on specific areas within the body with high spatial precision. This allows for the selective stimulation of tissues or implantable devices and the activation of triggered release systems [10,11,12]. Ultrasound, especially high-intensity focused ultrasound, generates high pressure and heat through cavitation: the implosion of gas bubbles or liquid droplets. Physical parameters of ultrasound (frequency, amplitude, duration, etc.) can be finely tuned to control the pressure and heat experienced by the target, actuating ultrasound-responsive DDSs without harming the surrounding tissue. Furthermore, ultrasound can initiate sonoporation, where ultrasonic waves create pores in cell membranes and tissue barriers to enhance the uptake of drugs [13,14] while avoiding undesirable tissue damage (with the exception of killing cells on purpose, such as in cancer therapy).

Recent research on ultrasound-responsive DDSs has primarily focused on microbubbles, nanodroplets and other nanocarriers [15]. Microbubbles are formed by lipids, surfactants, or polymers and contain gas or volatile liquid that cavitate under ultrasound and release their cargos [16]. Nanodroplets of volatile liquids such as perfluoro-compounds can be vaporized under ultrasound in a process called acoustic droplet vaporization (ADV), initiating drug release [17]. There are other nanocarriers composed of various materials such as silica and carbon nanotubes that trap air, allowing for cavitation and making the nanocarriers ultrasound-responsive [16]. Various self-assembled micro-/nanoparticles, including liposomes and block copolymer micelles, disassemble under ultrasound—another method for initiating drug release [18,19,20].

Hydrogels of hydrophilic polymers crosslinked via noncovalent or dynamic bonds are also ultrasound-responsive [21,22,23]. Mooney et al. reported a self-healing hydrogel network of crosslinked alginate with mitoxantrone release enabled when exposed to ultrasound [24]. In that same work, ionically crosslinked chitosan was exposed to ultrasound and demonstrated similar ultrasound-responsive drug release behaviors. More recently, hydrogels crosslinked through the Diels–Alder reaction were reported to release proteins upon ultrasound stimulation [21,25]. In addition, capsules consisting of a variety of polymers such as poly(lactic-*co*-glycolic acid) PLGA, crosslinked polyethylene glycol (PEG), and turmeric granules have been tested for ultrasound-triggered release [26,27,28,29].

Micro- and nanoparticles with a gold shell have also been investigated for sequestering molecular cargos and for their potential triggered release by ultrasound. One approach to fabricating the gold shell is sintering latex colloidosomes (capsules with shells composed of colloidal particles) plated with elemental gold [30]. These gold-plated colloidosomes retained a small hydrophilic drug (doxorubicin) but the ability of triggered release was not known [30]. Importantly, this method requires a sintering step, which is incompatible with thermo-sensitive cargos. A more amenable process of creating gold shells is electroless plating, which creates the protective gold shell entirely within the aqueous phase without a sintering step.

Electroless metal plating, or autocatalytic plating, is a chemical technique for producing metal-coated surfaces without an electrical current. Instead, electroless plating relies on the chemical reduction of metal ions onto a surface or a particle. This is particularly useful for applying small-scale platings to complex geometries, including nanoscale and microscale particles. Electroless plating has been used to create nanoscale, oil-filled gold shells to release a lipophilic dye in response to ultrasound [12]. The same research group further demonstrated that hexadecane-filled gold-plated capsules can release hexadecane, a hydrophobic alkane hydrocarbon of 255 Da, when triggered by clinically relevant low ultrasound intensities [31]. These gold-plated capsules can retain hexadecane on the order of days without leakage.

While these systems are very promising, the cargos released are all hydrophobic molecules, whereas many important drugs including biologics are hydrophilic. Furthermore, the core of these gold-coated microcapsules is either hollow or consists of hydrophobic, water-insoluble polymers such as polymethylmethacrylate (PMMA) and PLGA. Gold-coated hydrogel particles for the full retention and ultrasound-triggered release of hydrophilic molecules have not been reported [32,33,34].

**Figure 1:**
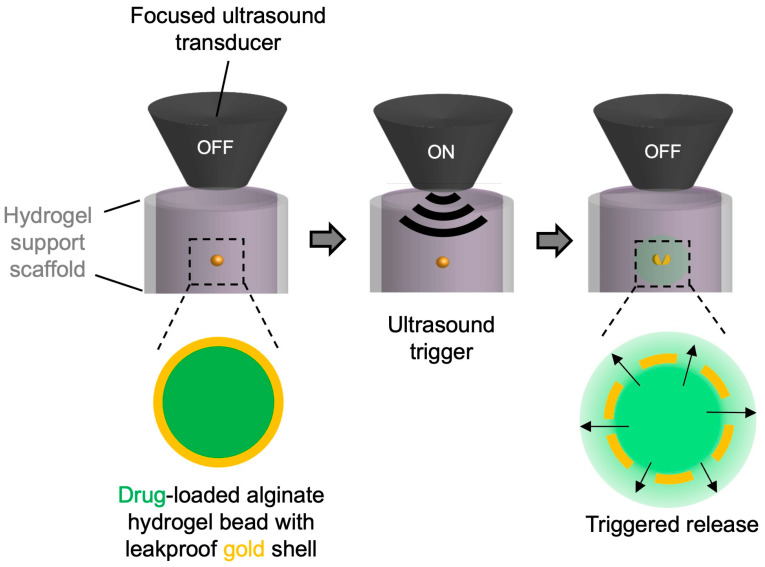
Schematic illustration of the general approach to ultrasound-triggered release of drugs from alginate hydrogel beads with leakproof gold shell.

Here, we present, to our knowledge, the first synthesis of a gold-plated hydrogel using electroless plating and the demonstration of a sharp off/on release of a difficult-to-sequester hydrophilic small molecule actuated by ultrasound (Figure 1). We selected calcium-crosslinked alginate hydrogel beads as the core, to which metallic nanoparticles were adsorbed to catalyze the electroless plating of the gold shell. This system leverages alginate as a biocompatible natural biopolymer widely used in many biomedical applications and provides a new tool for the remote control of the spatiotemporally defined release of bioactive molecules [35].

## 2. Materials and Methods

### 2.1. Materials

Alginic acid sodium salt from brown algae (ALG) (MW 240,000), Nile red, chloroauric acid (HAuCl_4_), chloroplatinic acid hydrate (H_2_PtCl_6_·H_2_O), ethylenediamine tetra-acetic acid (EDTA), sodium borohydrate (NaBH_4_) and polyvinylpyrrolidone (PVP) (MW 40,000) were all purchased from Sigma-Aldrich (St. Louis, MO, USA). Calcium chloride (CaCl_2_), DPBS (Gibco, 1X concentration), hydrogen peroxide (H_2_O_2_, 30% *v/v*), and fluorescein were purchased from ThermoFisher (Waltham, MA, USA). Polyethylenimine (linear MW 25,000) (PEI) and α-poly-L-lysine (MW 30,000) (PLL) were purchased from PolySciences (Warrington, PA, USA). All chemicals listed above were used without modification or purification.

### 2.2. Synthesis of Platinum Nanoparticles

Platinum nanoparticles (Pt NPs) were synthesized using an adapted method [33]. Briefly, H_2_PtCl_6_·6H_2_O (0.23 g) was added to 100 mL of 1.15 µM (0.0046 wt %) aqueous solution of PVP and stirred to dissolve. An aqueous solution of 0.5 mM NaBH_4_ (0.4 mL per 100 mL PVP solution) was then added to the preceding mixture and stirred vigorously for 2 min, reducing platinum from its salt form onto the solubilized PVP. The mixture darkened and was left to rest overnight. The formation and distribution of Pt NPs was characterized by dynamic light scattering (DLS) with the Brookhaven 90Plus Nanoparticle Size Analyzer (Brookhaven Instruments, Nashua, NH, USA) and assessed for batch-to-batch variability. Batches with particles ≤ 20 nm in size were then concentrated from 3 mL to 0.5 mL via centrifuge filtration with the Vivaspin^®^ 2 Centrifugal Concentrator (2000 MWCO) (Sartorius, Arvada, CO, USA) spun at 1200× *g* for 10 min. Concentrated samples were used within two months or frozen at −20 °C for long-term storage.

### 2.3. Preparation of ALG Beads

ALG (sodium salt) was dissolved in deionized (DI) water at 1.5 wt % by mixing at 300 RPM overnight. After ALG homogenization, fluorescein was dissolved in the alginate solution at 1 mg/mL. Subsequently, the ALG was extruded from a 22G needle dropwise into fluorescein-loaded (1 mg/mL) CaCl_2_ (10% *w*/*v*) spun at 60 RPM where it crosslinked into beads. After at least one minute to crosslink, ALG beads were then gently washed over a 70 μm cell strainer with DI water several times and placed into 5 mL centrifuge tubes.

### 2.4. Electroless Plating of ALG Beads

ALG beads were submerged in 4 mL of cationic solutions, either 0.1% *w*/*v* PEI solution or a 0.05% *w*/*v* PLL solution in DI water (adjusted to neutral pH), for five minutes on a carousel to make coated ALG/PEI beads and ALG/PLL beads, respectively. Polycation-coated ALG beads were gently rinsed once with DI water before being incubated with 2 mL solution of Pt NPs (0.5 mL concentrated particles and 1.5 mL DI water to a total volume of 2 mL) for 2 min on a carousel before a brief rinse with DI water. Finally, the beads were placed in 3 mL of a 1:3:1 volume mixture of 40 mM HAuCl_4_, 2% *w*/*v* PVP and 60 mM H_2_O_2_ for 1 to 20 min at room temperature, with a final rinse with DI water to terminate the plating process.

### 2.5. Scanning Electron Microscopy (SEM)

To examine the surface features of the ALG beads, SEM was performed using a Apreo 2S Lo-Vac electron microscope (Thermo Fisher Scientific, Waltham, MA, USA) operated at an accelerating voltage of 5 kV and an emission current of 50 pA, with 50 Pa of water vapor pressure. Prior to imaging, the beads were adhered to SEM specimen stubs by double-sided carbon tape. Samples were not dehydrated prior to imaging.

### 2.6. Determination of Gold Shell Thickness

Gold-plated ALG beads were embedded in Optimal Cutting Temperature (O.C.T.) Compound (Sakura Finetek USA, Torrance, CA, USA) and frozen at −20 °C overnight. Samples were sectioned into 10 μm thick slices using the cryotome Leica CM1990 Cryostat (North Central Instruments, Plymouth, MN, USA) and the slices were imaged on an Olympus IX-70 inverted microscope (Olympus America Inc., Waltham, MA, USA). Shell thickness was calculated using several points per section measured with ImageJ analysis software (version 1.54k).

### 2.7. Leakage Test

Crosslinked ALG beads with fluorescein loaded were placed in 1.7 mL conical tubes and covered with 1 mL of DI water. After 90 min at room temperature, the supernatant was retrieved and the intensity of fluorescence emission by fluorescein was measured using a Bio-TEK Cytation 3 plate reader (Agilent Technologies, Santa Clara, CA, USA) at an excitation wavelength of 498 nm and emission wavelength of 517 nm. The release fluorescein was quantified using a standard curve constructed using pure fluorescein solutions with known concentrations.

### 2.8. Focused Ultrasound-Triggered Release

Gold-plated ALG beads were exposed to focused ultrasound waves from one of two single-element immersion ultrasound transducers with a center frequency of 1 MHz (Olympus America Inc., Waltham, MA, USA Item # I8-0118-P-SU-F1.90IN or # A302S-SU-F1.63IN-PTF, Olympus America Inc., Waltham, MA, USA) driven by a 200 W amplifier (E&I 2200 L). Pulsed ultrasound carrier signals were delivered by a Keysight 33500B Series Waveform generator (Keysight Technologies, Loveland, CO, USA). The ultrasound transducer was focused using a custom-designed focusing cone filled with degassed water and coupled to the sample using clinical ultrasound gel. Gold-plated ALG beads were partially embedded in a 1% *w*/*v* ALG hydrogel to ensure that they did not shift outside of the focused ultrasound beam during the experiment. To create a scaffold for the gold-plated ALG, ALG gels were crosslinked over filter paper soaked in CaCl_2_ in 6-well plates to a minimum height of 5 mm. Elevating the platform above the bottom of the plastic well is important to reduce the magnitude of reflected ultrasound waves. Waves reflected off the bottom of the well may artificially increase gold rupturing, so elevation ensures that any rupturing is a result of the applied ultrasound alone and not a reflection of the experimental setup. A gold-plated ALG bead was placed onto a slice of alginate on top of the alginate scaffold, lightly covered with 50 μL of uncrosslinked 1% *w*/*v* ALG, followed by 200 μL of 10% CaCl_2_ pipetted along the bottom to adhere the gold-plated ALG to the scaffold. The remaining well volume was filled with water. Gold-plated ALG was exposed to ultrasound for 1 to 2 min using peak negative ultrasound pressures of 750 and 1000 kPa, a pulse width of 10 ms, and a pulse repetition period of 1 s. To achieve the above pressure levels, a hydrophone and water tank setup were used to directly measure the ultrasound output from the transducer for calibration before each experiment. The waveform generator amplitude was adjusted accordingly before each experiment to ensure accurate ultrasound pressure output, with typical waveform generator inputs of a peak-to-peak voltage of 75–130 millivolts (mVpp) into a 200 W amplifier to achieve the desired peak negative pressure amplitudes.

Immediately after ultrasound exposure, the top slice of the alginate hydrogel scaffold, including the embedded bead, was excised and transferred using tweezers to a separate container filled with a known volume of DI water. Fluorescein release post ultrasound triggering was determined by sampling the release medium at various time points and quantifying the fluorescein content by fluorimetry as described above in Section 2.6. To ensure reproducibility, two separate batches of the beads were measured for fluorescein release through experiments performed on different days. Each experiment involved three individual beads.

## 3. Results

### 3.1. Synthesis of ALG Beads, Pt NPs and Gold Shell

**Figure 2:**
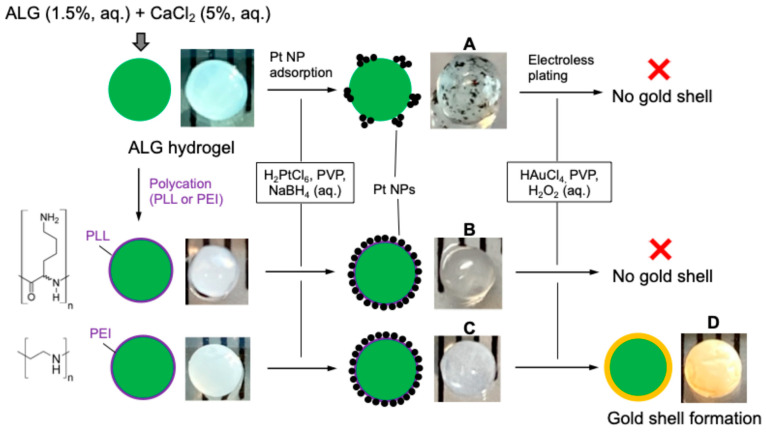
A schematic illustration of the pathways of the synthesis of gold-plated ALG hydrogel beads. Representative photos of the beads at key stages of the synthesis are shown. Catalytic Pt NPs were adsorbed to the surface of ALG beads without or with a precoated polycation (PLL or PEI) followed by electroless plating. Only the ALG beads precoated with PEI succeeded in forming a contiguous gold shell. (**A**–**D**) Photos of ALG beads at various stages of the synthesis.

The synthesis of gold-plated ALG hydrogel beads was attempted via three methods (Figure 2). ALG solution was extruded through a syringe needle into a CaCl_2_ bath and crosslinked into beads. For consistency, beads closest to 3 mm were selected for use in the subsequent experiments. The PVP-stabilized Pt NPs were synthesized by reducing a Pt salt to elemental Pt by NaBH_4_ (Supplementary Materials, Equation (S1) in Figure S1) in the presence of PVP (40 kDa) [33]. The DLS data indicate that the Pt NPs are sufficiently small and monodisperse at around 12 nm (Supplementary Materials, Figure S2). Effective Pt NP batches had sizes below 20 nm. There were no visible particles or aggregates to the naked eye and no settling at 4 °C or above when left unperturbed for several days. Batches of Pt NPs greater than 20 nm were also produced, but they often aggregated in solution and thus were not used in the subsequent experiments.

The Pt NPs must adsorb to the surface of the ALG beads to catalyze the formation of a gold shell via electroless plating. Unfortunately, PVP-stabilized Pt NPs formed sparse patches of highly aggregated, large clusters on the surface of the ALG beads (Figure 2, photo A) and failed to catalyze the reduction of HAuCl_4_ by H_2_O_2_ (Supplementary Materials, Equation (S2) in Figure S1). To facilitate the adsorption of Pt NPs, the negatively charged ALG beads were first precoated with a polycation. Two cationic polymers were explored: PLL and PEI. As expected, Pt NPs adsorbed to both types of precoated ALG beads, judging by the change in appearance from clear to translucent to slightly opaque and gray (Figure 2, photos B and C). Surprisingly, the subsequent electroless plating of the PLL-coated ALG beads failed to form a gold shell, whereas the PEI-coated beads turned yellow and maintained a smooth surface texture without visible defects, indicating the formation of a uniform and contiguous gold shell (Figure 2, photo D).

### 3.2. Microscopic Morphology and Thickness of the Gold Shell

**Figure 3:**
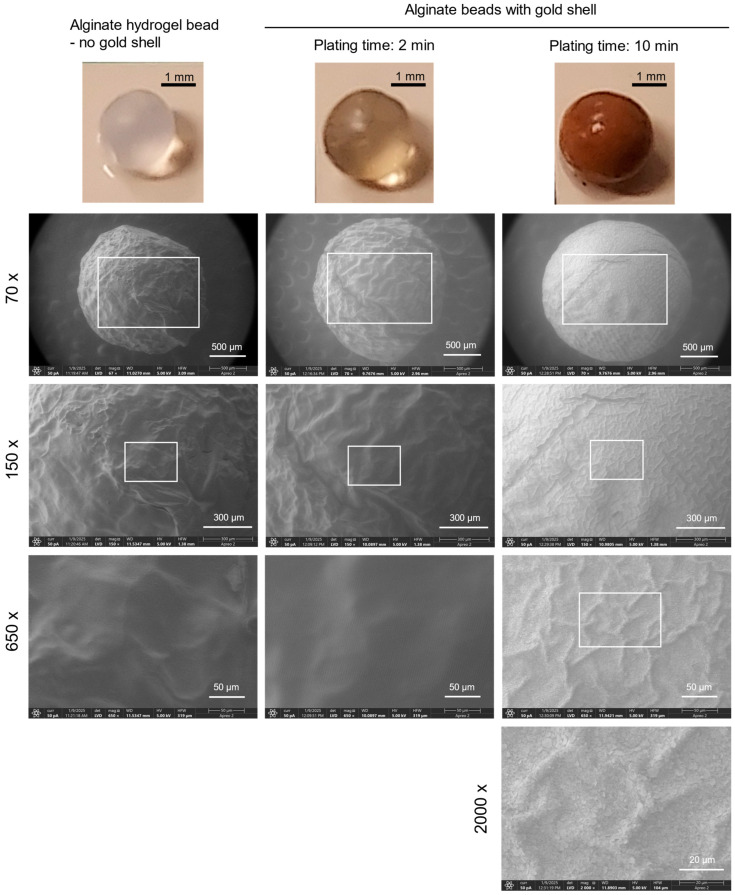
Visible light photographs and SEM images of ALG hydrogel beads with or without the gold shell. The gold shell was formed after either 2 min or 10 min plating.

ALG hydrogel beads with or without the gold shell were photographed under ambient lighting. A longer plating time resulted in beads with a darker color, indicative of a thicker gold shell (Figure 3). To observe the microscopic morphology of the gold shell, SEM images were captured for the ALG hydrogel bead with or without a gold shell. Although the SEM operated with low vacuum, the ALG bead without a gold shell showed significant shrinkage due to dehydration, as expected for a hydrogel with high water content (Figure 3). The ALG bead with a gold shell formed by 2 min of plating also appeared shrunken with a rough surface and many wrinkles. However, the bead with 10 min plating experienced some shrinkage but maintained its round shape and a much smoother surface, presumably due to a thicker and stiffer gold shell, and high-resolution SEM (2000× magnification) revealed the presence of gold grains on the surface.

**Figure 4:**
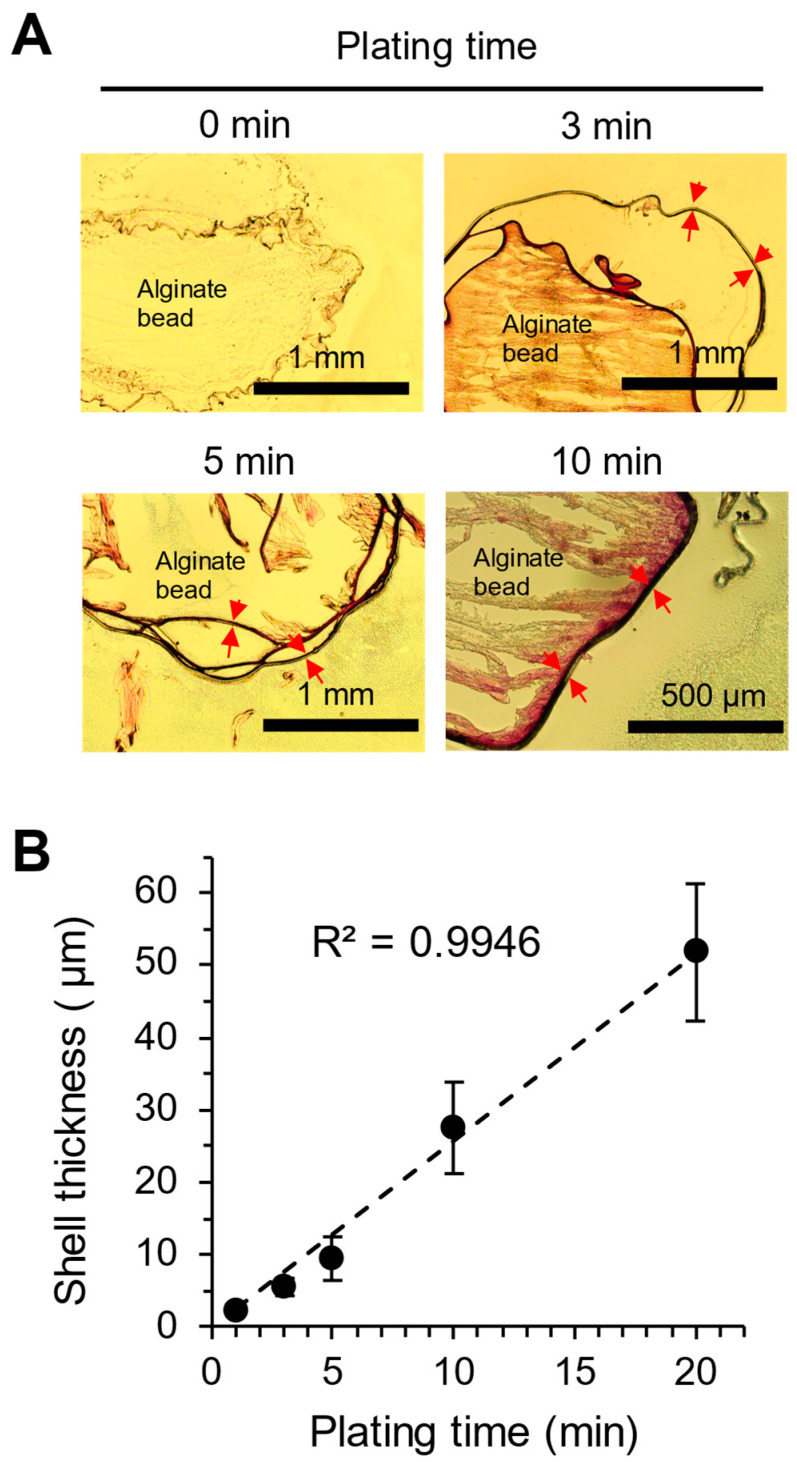
Thickness of the gold shell as a function of electroless plating time. (**A**) Representative light microscopy images of the cross-section of gold-plated ALG hydrogel bead at various plating times. Red arrows in A indicate multiple locations of the shell where the thickness is measured. (**B**) A quantitative correlation between gold shell thickness and plating time. The dashed line is a linear fit of the data, which are the means ± SD of at least 5–20 measurements.

To determine the thickness of the gold shell, the ALG hydrogel beads were cryosectioned into 10 μm thick slices. Brightfield light microscopy images of the cross-section reveal the formation of a gold shell as dark stripes that become thicker with a longer plating time (Figure 4A). The shells appear to be contiguous, largely uniform in thickness without defects. At longer plating times, the interior of the ALG hydrogel acquired a pink color due to the reduction of small amounts of Au^3+^ ions by H_2_O_2_ having diffused into the hydrogel during plating. Sometimes, multiple strands of the gold appear to lay on top of each other (for example, Figure 4A, the 5 min sample). This is because parts of the shell fracture during sectioning and the resulting fragments fold upon themselves. The ALG hydrogel also appears fractured and often detached from the shell, suggesting a mechanical mismatch between the soft hydrogel and the rigid metallic shell. Using images, the thickness of the shell was measured at multiple locations, averaged and plotted against the duration of plating from 1 to 20 min, showing a very good linear correlation (Figure 4B). A 10 min plating time was selected to prepare gold-plated ALG beads for the leakage and triggered release studies below.

### 3.3. Leakage Test

**Figure 5:**
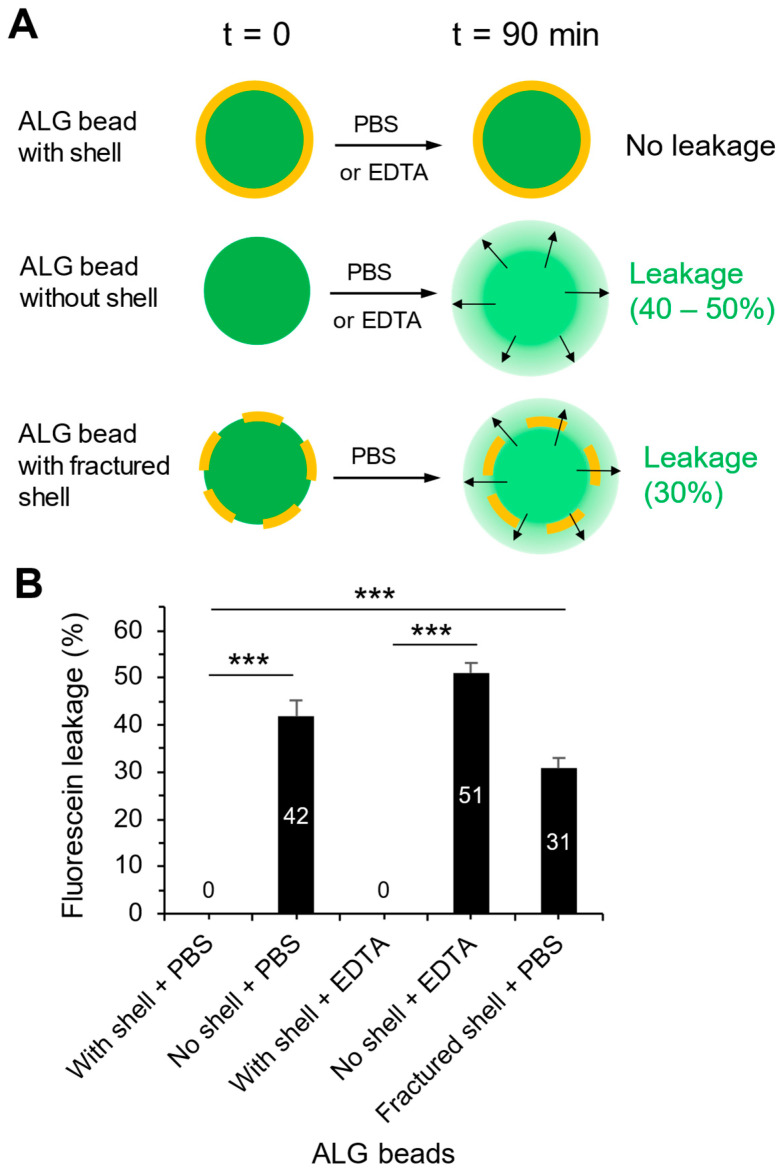
Verification of the gold-plated ALG hydrogel beads with a leakproof shell. (**A**) Illustration of the leak test and its outcome. (**B**) Quantification of fluorescein leakage. Data representing mean ± SD. ***: *p* < 0.001, Student’s *t* test (unpaired) and one-way ANOVA with Tukey–Kramer post hoc analysis, *n* > 3 samples, with each sample measured in triplicate.

Gold-plated ALG hydrogel beads were loaded with fluorescein, a water-soluble small fluorophore (MW 332 Da), and assessed for leakage by immersion for 90 min in aqueous media. There was zero leakage of fluorescein from the ALG beads immersed in either PBS alone or with EDTA (MW 292 Da), a chelator that would dissolve the Ca^2+^-crosslinked ALG core if it could diffuse through the gold shell (Figure 5A). In contrast, ALG beads without the gold shell leaked out 42% of the fluorescein after 90 min in PBS and lost 51% in the presence of EDTA while showing obvious swelling and signs of disintegration. The fluorescein-loaded gold-plated beads were stored in aqueous buffer for up to eight months at room temperature. No leakage of the fluorescein into the buffer was detected. These observations suggest that the gold shell is an effective barrier to prevent the bilateral diffusion of water-soluble molecules as small as ~300 Da. To further prove this point, when the gold-plated ALG beads were fractured manually to break the gold shell, the leakage of fluorescein was 30% after 90 min in PBS. The difference in the amount of leakage is highly significant between the beads with an intact shell and those without a shell or with a fractured shell (Figure 5B).

### 3.4. Focused Ultrasound Experimental Setup and Pressure Mapping

**Figure 6:**
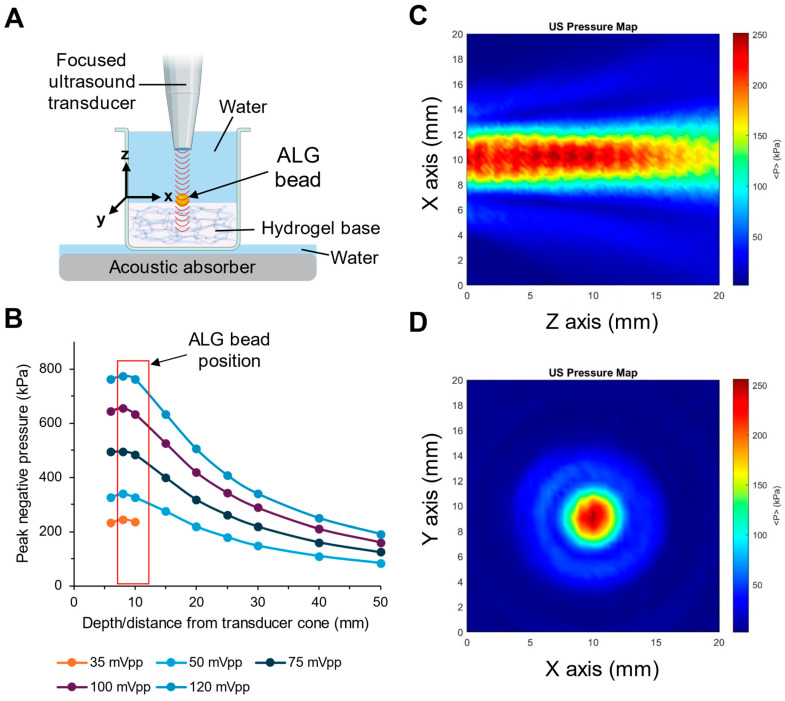
Focused ultrasound experimental setup and ultrasound-generated spatial pressure profiles. (**A**) Graphic depiction of the experimental setup. (**B**) Ultrasound peak negative pressure profile produced by a range of waveform generator peak to peak output voltages. (**C**,**D**) Spatial mapping of the ultrasound beam pressure distribution measured beginning at the tip of the transducer fitted with a focusing cone using a waveform generator input of 45 mVpp. Panel (**D**) is a cross-section recorded at the depth of maximum peak negative pressure.

Gold-plated ALG beads were mounted onto an ALG hydrogel base and secured with a thin layer of a rapidly crosslinked ALG. The hydrogel base provided physical support and kept the beads immobile during the ultrasound stimulation (Figure 6A). Prior to placing the ultrasonic transducer on the hydrogel, the ultrasound peak negative pressure was measured as a function of the distance from the tip of the focusing cone, and various input levels from the waveform generator were assessed in a water tank using a calibrated hydrophone. The peak pressure attenuated at distances greater than 10 mm from the tip of the focusing cone (Figure 6B). Therefore, when positioning the transducer and cone, a distance of 10 mm above the gold-plated ALG beads was used so that the beads would experience the maximum pressure. Two-dimensional pressure mapping shows that if a bead 3 mm in diameter is positioned directly under the transducer and cone at a distance of 10 mm, it will be within the range of the maximum pressure (Figure 6C,D). To further reduce the magnitude of reflected ultrasound waves and ensure an accurate ultrasound magnitude, we coupled an acoustic absorbing rubber (Aptflex28, Precision Acoustics Ltd., Dorchester, Dorset, UK) to the underside of the plate using water.

### 3.5. Ultrasound-Triggered Release

**Figure 7:**
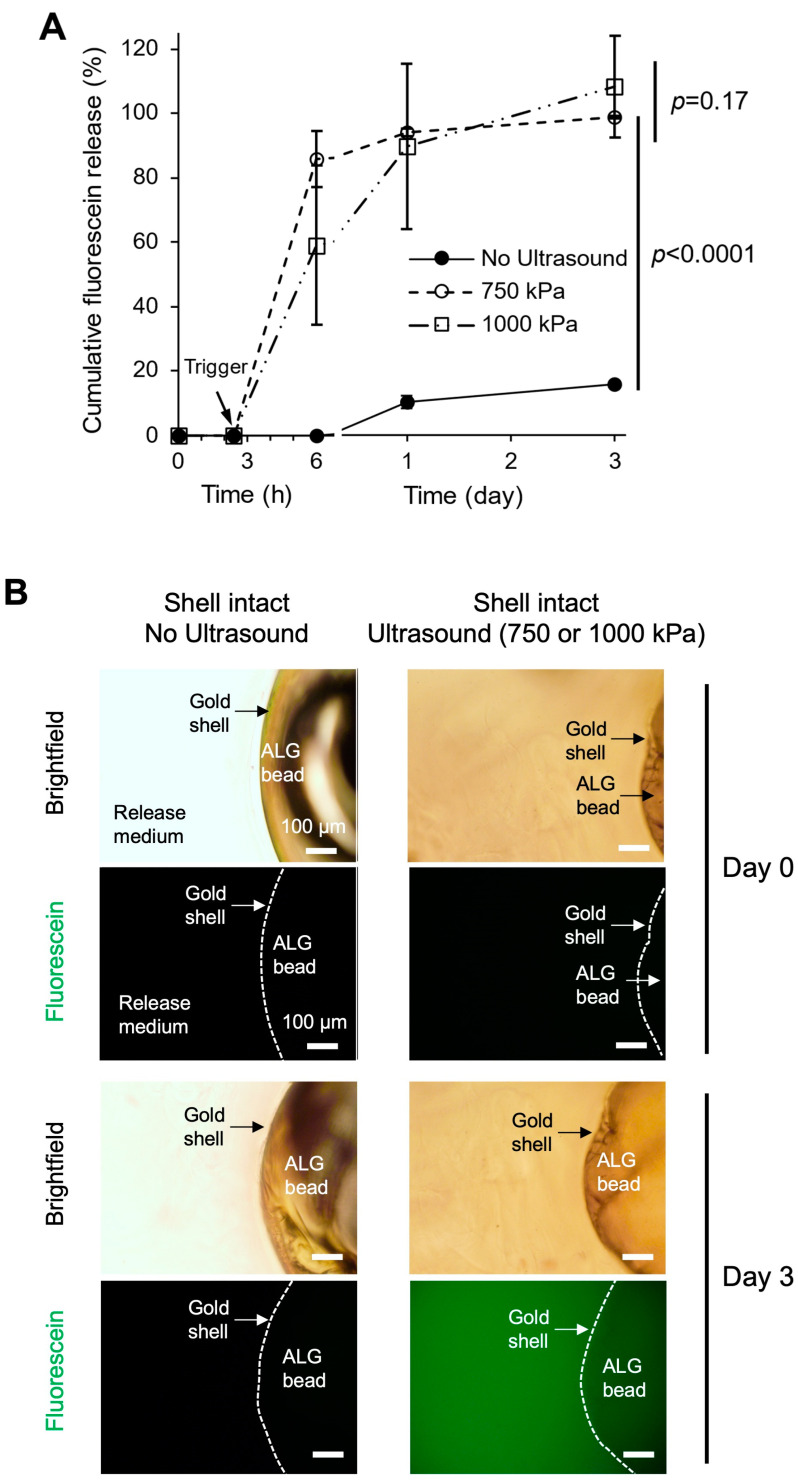
Focused ultrasound-triggered release of fluorescein from gold-plated ALG beads. (**A**) Cumulative release of fluorescein with or without ultrasound trigger over 3 days. The duration of ultrasound actuation is 2 min. Data are the average of 3 different beads from each of the two separate batches. Unpaired *t* test, two-tailed *p* values calculated for two-group comparisons. (**B**) Representative brightfield and fluorescence microscopy images of the beads and the release medium taken on day 0 and day 3 before and after ultrasound-triggering.

Gold-plated ALG beads loaded with fluorescein were exposed to 2 min of focused ultrasound in water. The samples were then immediately transferred to well plates containing water to quantify the cumulative fluorescein release and be observed under a fluorescence microscope for 3 days. Beads exposed to focused ultrasound showed over 50% release within the first two hours and 100% release by day 3 (Figure 7A). There was no significant difference in the release between 750 and 1000 kPa pressures of focused ultrasound. Comparatively, samples not exposed to ultrasound showed no release in the first 2 h and a small amount of release after 24 h due to damage to the gold shell by handling with a tweezer and from fixation into the hydrogel scaffold. These quantitative results are corroborated by visual inspection using fluorescence microscopy, showing a significant increase in the green fluorescence signal (from fluorescein) in the vicinity of the gold-plated beads after ultrasound triggering (Figure 7B).

## 4. Discussion

### 4.1. Design Rationale of the Ultrasound-Triggerable Gold-Plated ALG Hydrogel

For effective integration into research and therapeutic settings, an ideal delivery system should enable on-demand cargo release, minimizing or eliminating leakage in the absence of a triggering stimulus. In our study, we employed focused ultrasound, a system with proven clinical usage, to demonstrate the feasibility of our DDS to achieve externally triggered release [36]. We chose to use crystalline gold as the barrier to sequester our cargo because gold is well known for its inertness and biocompatibility [37]. We chose to use ALG to form the core for the gold shell because it is a biocompatible, hydrophilic biopolymer, easily and reversibly crosslinkable, and extensively studied for numerous biomedical applications [35]. As proof of principle, we loaded ALG hydrogel beads with fluorescein, a water-soluble, rapidly diffusing fluorophore, as a model drug to be sequestered in gold-coated ALG hydrogel and investigated its release triggered by ultrasound (Figure 1).

Electroless plating, also known as autocatalytic plating, involves the deposition of a layer of metal ion by ion onto a surface without the application of an electrical current [38]. This technique is particularly advantageous for achieving a uniform metal shell thickness on substrates of complex geometries. Further, it can be applied to electrically inert materials and surfaces. Previous studies have confirmed the feasibility of electroless plating on micro- and nanoscale particles consisting of organic liquids or hydrophobic polymers dissolved in organic solvents [32,33]. However, electroless plating has not been applied to hydrophilic substrates such as hydrogels. Here, for the first time, we report the synthesis of gold-plated hydrogel particles by electroless plating and demonstrate the utility of this novel material to sequester and release small water-soluble cargo upon actuation by ultrasound.

### 4.2. Optimization of the Methodology of Synthesis

First, the ALG hydrogel beads of millimeter sizes were formed by extruding ALG aqueous solution through a syringe needle followed by crosslinking by Ca^2+^. We intentionally chose a large size for the ALG beads because millimeter-size particles of ALG implanted in animals have shown a much reduced foreign body response and inflammation than smaller beads irrespective of their stiffness [39]. Fluorescein was added to both the CaCl_2_ bath and the ALG solution at 1 mg/mL to avoid the loss of loaded fluorescein due to diffusion into the Ca^2+^ bath during crosslinking. A typical ALG bead 3 mm in diameter has a volume of approximately 14 μL and is loaded with 14 μg fluorescein. In our experimental setup, the ALG bead is embedded partially within a hydrogel scaffold immersed in physiological buffer at 37 °C, mimicking the scenario of the bead implanted in a soft tissue environment and releasing its cargo triggered by focusing the ultrasound wave through an external transducer positioned in its proximity (Figure 1).

Next, we examined the impact of the size of Pt NPs on the outcome of the electroless plating of ALG beads. Here, the Pt NPs are synthesized by the NaBH_4_-mediated reduction of Pt^4+^ to colloidal Pt^0^ stabilized by PVP (40 kDa) in water (Figures S1 and S2). The size of the Pt NPs is controlled by the molar ratio of NaBH_4_ to H_2_PtCl_6_ [33]. An optimal PVP/Pt molar ratio (0.0046 wt %) is chosen because it is high enough to stabilize the Pt NPs and low enough to minimize potential competitive adsorption to the ALG beads by any free PVP in excess, per prior studies from which we adapted this protocol [33,40]. We have synthesized several batches of the Pt NPs of various sizes. For successful gold plating, we observed that the average diameter of the Pt NPs needed to be ≤20 nm. When Pt NPs exceeded 20 nm in diameter, they exhibited poor adhesion to surfaces and were inclined to form separate nodules of reduced gold rather than a contiguous layer of gold on the surface of ALG beads, similar to what we see in Figure 2A. As these larger particles also precipitate out of solution over time, we suspect that particle–particle aggregation is competing with particle–surface interactions, thus not being able to form a high-quality uniform gold shell. Prior works have achieved Pt NPs as small as 2 nm [33]. While we have also achieved similar sizes, for the triggered release data presented here, our particles were ~12 nm. To our knowledge, an upper size limit for this electroless plating technique has not been demonstrated in the literature until this work identified it to be 20 nm.

The final step of electroless plating is the reduction of Au^3+^ salt by H_2_O_2_ to elemental gold (Figure S1), resulting in the formation of a contiguous gold shell around the ALG beads. This outcome depends on the adsorption of the catalytic Pt NPs onto the surface of the ALG beads. Unfortunately, PVP-stabilized Pt NPs could not adsorb properly but aggregated into sparse, large clusters and the gold shell failed to form (Figure 2). To facilitate the adsorption of Pt NPs, the negatively charged ALG beads were treated with a polycation. This idea is inspired by previous studies using a cationic surfactant cetrimonium bromide (CTAB) to treat the surface of polymethyl methacrylate (PMMA) particles to enable Pt NP adsorption and gold plating [32,34]. In these studies, the core material (i.e., PMMA or PLGA) was first emulsified in oil/water mixed solvents and then stabilized by PVP/Pt NPs adsorbed at the oil/water interface, forming Pickering emulsions [32,33,34]. Our ALG beads, however, are too large to form stable emulsions and therefore require the assistance of a polycation to enable Pt NP adsorption. We evaluated two polycations: PLL, a classic polymer used in combination with ALG for cell microencapsulation first reported in 1980, and PEI, widely used in the nonviral delivery of nucleic acids [41,42]. To our surprise, ALG beads pretreated with PLL could not form any gold shells, but PEI treated ALG beads formed a contiguous gold shell without an apparent defect (Figure 2). We speculate that the different outcome of the electroless plating of ALG beads pretreated with these two polycations may be due to multiple factors. PLL contains exclusively primary amines, whereas PEI contains both primary and secondary amines (see chemical structures in Figure 2). Compared to PEI, PLL is more densely charged under neutral pH and known to cause the flocculation of metal nanoparticles [43]. Flocculated Pt NPs may not properly adsorb to ALG beads and not able to form a contiguous gold shell. Furthermore, while the highly charged PLL primarily engages in ionic bonding through the primary amines, PEI is also able to hydrogen bond with the PVP through the secondary amines. The difference in secondary bonding may result in more uniform, less clustered, potentially single layers of the Pt NPs to PEI-treated ALG, which appeared to have a lighter gray color than the PLL-treated ALG (photos B and C, Figure 2). This speculation is supported by a literature report that less aggregated single-layer Pt NP coverage of particle surfaces leads to more uniform and thinner gold shells [32]. Future investigation is warranted to uncover the exact reason for the different outcome between PLL and PEI.

Pt-NPs have been shown to have potential as anti-cancer particles, with minimal impact on non-cancerous cells [44]. At concentrations below 10 μg/mL, it has been shown that PVP-stabilized Pt NPs 10 nm in diameter do not cause significant hemolysis [45]. It has also been shown that cancerous cells exposed to 6 μg/mL of Pt NPs have lower viability after 16–124 h, depending on the cell line. Given the very low presence of Pt NPs in the final ALG beads and their electrostatic interactions with the PEI, we do not expect cytotoxic behavior from the Pt NPs.

We considered the biocompatibility of gold with a survey of the literature. Gold does not stimulate significant acute cytotoxic effects from large-scale implants. However, toxicity has been observed with gold microparticles, though the effect is highly dependent on shape [46,47]. An analysis of metallic debris and its cytotoxic effects would be required to understand how gold shell rupture would affect local cells or tissues. A sterile synthesis and cell exposure assay will be important prior to in vivo use.

### 4.3. Controlling the Thickness of the Gold Shell

The thickness of the gold shell is directly proportional to the total quantity of gold that the catalytic surface is exposed to, which is determined by a combination of the gold ion concentration and the time for plating [33]. The size of the Pt NPs is not related to the gold layer’s thickness. In this work, we controlled the amount of gold available for plating by limiting exposure time to a solution of Au^3+^ ions. In line with the current literature [31,32,33], since the concentration of the HAuCl_4_ solution is fixed, the thickness of the gold shell is proportional to the length of the plating time with 1 min plating yielding thin film-like gold layers and 10 min plating times creating thick layers of gold (Figure 4). These results suggest a simple and efficient way of controlling the thickness of the gold shell using a plating solution with a fixed composition. Thicker shells may be stronger mechanically and could temper the shrinkage of the beads under the vacuum of the SEM (Figure 3).

### 4.4. Evaluation of the Permeability of the Gold Shell

The functional quality of the gold shell is that it should maintain near-zero permeability of the encapsulated cargo in an aqueous medium in the absence of an ultrasound trigger. To assess this, we performed a quantitative fluorescein leakage test in either phosphate buffer or in the presence of EDTA (a chelator to dissolve the crosslinked ALG gel) for 90 min (Figure 5). This test was designed to allow a relative quick assessment of leakage so we could screen a variety of material constructs and media conditions. For reference, nearly 50% of the loaded fluorescein leaked out of the ALG beads without gold shell protection. However, with the gold shell, the leakage of fluorescein was essentially zero. Only when the gold shell was fractured mechanically, about 30% fluorescein leakage was detected. These results demonstrate the excellent quality of the gold shell as a diffusion barrier for a small hydrophilic molecule in aqueous media. Importantly, the long-term retention of fluorescein was demonstrated after storing our beads in water for nearly 8 months at room temperature. This leakproof performance of our gold-plated ALG beads compares favorably with the research literature on other triggerable gold-plated particles containing various cargos such as hexyl salicylate, Dil, paclitaxel and hexadecane, which reported no leakage over 17 h to 10 days [12,34,48]. The extraordinarily long retention of cargo by our beads may be attributed to their relatively large particle size (thus low surface area) and nearly 30 μm thick gold shell (thus less permissive to molecular diffusion). Practically, our drug-loaded leakproof beads may have a long shelf life, a key requirement for a successful controlled release product.

### 4.5. Factors Influencing the Triggered Release by Ultrasound

In our ultrasound-triggered release experiments, a gold-plated ALG bead is centered underneath the focused ultrasound transducer in the XZ plane and then raised by 10 mm along the Z axis (Figure 6A). Moreover, 10 mm is chosen to be the distance from the transducer as the beam profile of the transducer shows pressure intensities in the 95th percentile at (10 mm, 10 mm) in the XZ plane (Figure 6B,C). Pressure maps were re-characterized prior to each experiment, and the beam profile was consistent between the experiments reported here.

We used a hydrogel support scaffold to embed the gold-plated ALG beads because not only it provides the stable immobilization of the beads but also allows for more efficient shell rupture than aqueous liquid media [12]. After being ultrasound-triggered, the beads along with the hydrogel scaffold were excised and transferred to a separate container for quantifying the release of the cargo. A potential issue with this setup is that the beads will have to be handled and transferred manually and subject to unintended mechanical damages, which may have resulted in a 20% baseline release by day 3 from the no-ultrasound samples (Figure 7A). However, the rapid triggered release after ultrasound stimulation is obvious, increasing from 0% to 50–80% release in 3 h. By day 3, beads stimulated by ultrasound at both 750 and 1000 Pa pressures achieved 100% release. This sharp off–on triggered release kinetic profile is superior to other triggerable gold-coated particles reported in the literature. For example, ultrasound triggering increased the release of Dil by 30% by day 3, 50–60% by day 7, and only 50–60% by day 35 [12]. Another study reported 30% triggered release for paclitaxel and 50% triggered release for hexadecane [48]. Being able to achieve high sensitivity to ultrasound stimulation, highly rapid release beyond a low baseline leakage and the near-100% complete release of the encapsulated cargo are significant advantages of our gold-plated ALG beads reported here.

We then attempted to further understand the mechanism behind the superior responsiveness of our beads toward ultrasound stimulation. When using ultrasound to initiate buckling in core–shell capsules, the required stimulus intensity is proportional to the ratio between the capsule’s diameter and the shell thickness, as previously reported [49,50]. The relationship can be described by the following equation:

$$P_{buckling}=3E\left(\frac{d}{R}\right)e_{x}$$

where *P_buckling_* is the external pressure exerted to cause shell buckling (i.e., the pressure applied through ultrasound stimulation and, in our case, 750 and 1000 kPa), *E* represents the Young’s modulus of the capsule shell material (79 GPa for bulk crystalline gold), an inherent material property, *d* denotes shell thickness (5.3 μm on average for our gold shell, according to Figure 4B), *R* indicates capsule radius (1.5 mm for our ALG beads), and *e_x_* signifies the critical strain, after which release from the aqueous system is inevitable [51]. Using this equation, we calculated the *e_x_* of our ALG beads to be 0.18% and 0.24% under 750 and 1000 kPa pressure, respectively. These are very small strains. For context, ultrasound-sensitive microcapsules of the biodegradable poly(L-lactide) (PLLA) had an *e_x_* of 1.8% and required a peak pressure of 7.54 MPa to buckle [50]. In comparison, the gold-plated ALG beads would buckle under one tenth of the strain under one tenth of the peak pressure of that of the PLLA particles. Being able to buckle under small strain and low peak pressure are desirable properties of an ideal ultrasound sensitive barrier material. In this sense, stiff metallic barriers such as gold are preferred over softer polymeric barriers. Moreover, while our ultrasound actuation operated with low peak pressures, we applied high numbers of cycles of stimulation within a 2 min span to further promote the buckling of the gold shell with a clinically relevant ultrasound transducer to output a range of ultrasound intensity within the FDA restrictions on peripheral vessel acoustic exposure levels [52]. With further study, we may be able to relate the ratio of shell to diameter with peak pressures to determine minimal compatible intensities of ultrasound for a given core–shell system.

## 5. Conclusions

Our study demonstrates the first successful synthesis of gold-plated hydrogels by electroless plating and their application for ultrasound-triggered drug release. This system combines the biocompatibility and versatile drug-loading capacity of ALG hydrogels with an ultrasound-rupturable gold shell. Our results reaffirm that ultrasound can effectively trigger the release of encapsulated small molecule drugs, highlighting the potential of this technology for precise, on-demand drug delivery. This advancement not only broadens the scope of ultrasound-triggered release systems beyond hydrophobic compounds and organic oil phases but also opens new possibilities for the delivery of hydrophilic therapeutics that utilize the hydrogel core as a depot to achieve variable release kinetics. Applications include the on-demand release of protein-based therapeutics, which are rapidly gaining traction amongst pharmaceutical companies, and stem cell-based in vitro studies with precise spatiotemporal release requirements [53]. Many protein drugs are highly potent with a narrow therapeutic window, therefore requiring tight control over the timing of release and precise dosing to prevent off-target side effects. For stem cell-enabled regenerative medicine and tissue engineering, morphogens that drive cell differentiation are also highly potent and act transiently to drive tissue formation. The action of these cell fate determinants must be tightly regulated to be directed to specific cell populations for the appropriate duration. Our ultrasound-triggerable hydrogel is a new addition to the toolkit of on-demand drug delivery systems and may be particularly suited for highly potent protein-based therapeutics and morphogens for stem cell modulation. Future work will focus on the high-throughput fabrication of microgels, optimizing the thickness of the gold layer (to minimize the required ultrasound pressure/duration) and exploring the release of biologically relevant agents in in vivo studies.

## Data Availability

The original contributions presented in the study are included in the article, further inquiries can be directed to the corresponding author.

## Supplementary Materials

The following supporting information can be downloaded at: https://www.mdpi.com/article/10.3390/pharmaceutics17010133/s1, Figure S1: The chemical reduction of Pt salt (Equation (S1)) and ionic Au (Equation (S2)) in water; Figure S2: Size distribution of the PVP-stabilized Pt NPs used in the electroless plating of gold shell onto the ALG hydrogel beads. Measurements were made in deionized water by dynamic light scattering (DLS).
