# Supplementary material for: Alginate Hydrogel Beads with a Leakproof Gold Shell for Ultrasound-Triggered Release
Source: Pharmaceutics. 2025 Jan 19;17(1):133. doi: 10.3390/pharmaceutics17010133 (PMC11768098; doi:10.3390/pharmaceutics17010133)
Supplement: Supplementary file 1 [file pharmaceutics-17-00133-s001.zip › pharmaceutics-3419279-supplementary.pdf]

Supplementary Materials

$$NaBH_4 + H_2PtCl_6 + 3H_2O \rightarrow Pt + H_3BO_3 + 5HCl + NaCl + 2H_2 \quad \text{(Equation (S1))}$$

$$2HAuCl_4 + 3H_2O_2 \xrightarrow[Pt]{} 2Au + 3O_2 + 8HCl \quad \text{(Equation (S2))}$$

Figure S1. The chemical reduction of Pt salt (Equation (S1)) and ionic Au (Equation (S2)) in water. (Adapted from Stark 2019 [31])

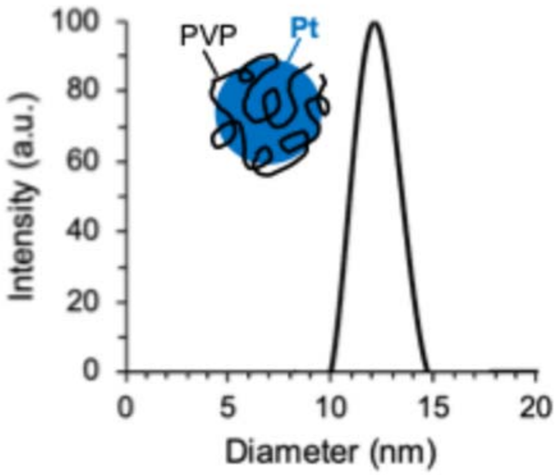

Figure S2. Size distribution of the PVP-stabilized Pt NPs used in the electroless plating of gold shell onto the ALG hydrogel beads. Measurements were made in deionized water by dynamic light scattering (DLS).
